# The effect of personalised versus non-personalised study invitations on recruitment within the ENGAGE feasibility trial: an embedded randomised controlled recruitment trial

**DOI:** 10.1186/s12874-022-01553-5

**Published:** 2022-03-06

**Authors:** Ella Thiblin, Joanne Woodford, Mattias Öhman, Louise von Essen

**Affiliations:** 1 Healthcare Sciences and e-Health, Department of Women’s and Children’s Health, Uppsala University, Dag Hammarskjölds väg 14B, 751 05 Uppsala, Sweden; 2 Institute for Housing and Urban Research, Uppsala University, Uppsala, Sweden

**Keywords:** Recruitment, Retention, Study invitation, Study within a trial, Trial methodology, Randomised controlled trial

## Abstract

**Background:**

Recruitment into clinical trials is challenging and there is a lack of evidence on effective recruitment strategies. Personalisation of invitation letters is a potentially pragmatic and feasible way of increasing recruitment rates at a low-cost. However, there is a lack of evidence concerning the effect of personalising of study invitation letters on recruitment rates.

**Methods:**

We undertook a Study Within A Trial (SWAT) to investigate the effect of personalised versus non-personalised study invitation letters on recruitment rates into the host feasibility trial ENGAGE, a feasibility study of an internet-administered, guided, Low Intensity Cognitive-Behavioural Therapy based self-help intervention for parents of children previously treated for cancer. An intervention group (*n* = 254) received a personalised study invitation letter and the control group (*n* = 255) received a non-personalised study invitation letter. The primary outcome was the proportion of participants in the intervention group and the control group enrolled into the ENGAGE host feasibility trial. Secondary outcomes relating to the recruitment and screening process, and retention were examined. Differences in proportions between groups for the primary and secondary outcomes were estimated using logistic regression.

**Results:**

Of the 509 potential participants, 56 (11.0%) were enrolled into the ENGAGE host feasibility trial: personalised: 30/254 (11.8%) and non-personalised: 26/255 (10.2%). No statistically significant effect on personalisation of enrolment was found (OR 1.18, 95% CI 0.68–2.06). No statistically significant differences were found for any secondary outcome.

**Conclusions:**

Personalisation of study invitations had no effect on recruitment. However, given the small study sample size in the present SWAT, and lack of similar embedded recruitment RCTs to enable a meta-analysis, additional SWATs to examine the personalisation of study invitation letters are warranted.

**Trial registration:**

ISRCTN57233429; ISRCTN18404129; SWAT 112, Northern Ireland Hub for Trials Methodology Research SWAT repository (2018 OCT 1 1231) (https://www.qub.ac.uk/sites/TheNorthernIrelandNetworkforTrialsMethodologyResearch/FileStore/Filetoupload,939618,en.pdf).

**Supplementary Information:**

The online version contains supplementary material available at 10.1186/s12874-022-01553-5.

## Background

Randomised controlled trials (RCTs) are generally considered the gold standard for evaluating healthcare interventions, but often face challenges with the recruitment [1, 2] and retention [3] of participants. Extended recruitment periods, failure to reach recruitment targets [2, 4], and poor retention [3] are common, resulting in poor research quality and monetary loss [2, 4]. Low recruitment and retention rates also lead to insufficient statistical power, increasing the risk of either type I (a false positive finding) or type II (a false negative finding) errors [5]. There are also ethical considerations with participants investing time and energy in trials that might not generate results that can adequately answer the research question [4]. Highlighting these difficulties, recent reviews of trials conducted in the United Kingdom between 2002 and 2016 found only 55–56% reached their original recruitment target [2, 3]. Given these challenges, the need for trial methodology research to improve trial process efficiency is clear [6, 7]. The conduct of studies within a trial (SWATs) (i.e., a study embedded within a host trial aimed to evaluate trial processes, such as recruitment and retention) is a way to establish such an evidence base, and hopefully lead to reduced research waste [7, 8]. Emphasising the importance of research to improve trial process efficiency, multiple initiatives for prioritising research to evaluate trial processes have been undertaken, such as the Medical Research Council funded Techniques for Assisting Recruitment to Trials (MRC-START) programme [9]; Prioritising Recruitment in Randomised Trials (PRioRiTy)-study [10]; the Trial Forge Platform [11]; and the Online Resource for Research in Clinical triAls (ORRCA) project [12].

Despite trial recruitment and retention being common challenges, recent Cochrane reviews have identified a lack of evidence concerning strategies to improve recruitment [4] and retention [13]. Previous research has shown that adopting an open trial design (e.g., participants know which intervention they will receive), and telephone reminders during the recruitment phase increase recruitment rates [4]. However, other strategies have produced varying effects. For example, a recent meta-analysis investigating the effect of user-tested, simplified, and clarified study information sheets on recruitment rates showed no effect [14]. Monetary incentives were found to be effective in one trial [15], whereas access to video clips with study information online [16, 17] was not. However, currently there are too few studies examining each strategy for conclusions to be drawn and our understanding of how to recruit effectively to trials is limited [4, 18]. One potential way of optimising recruitment is the personalisation of trial documentation, with a systematic review suggesting personalisation can improve questionnaire return rates [19]. However, this review included a wide variety of personalisation strategies, for example, signing letters personally and hand-addressing envelopes making it difficult to know which personalisation strategy/ies may have a positive effect on recruitment [20]. Further, the current literature has predominantly focused on returning questionnaires or surveys [21–23] and very few studies utilising a RCT design, have been conducted within the context of healthcare intervention research, to examine the personalisation of study invitations on recruitment rates [24]. Indeed, in the latest Cochrane systematic reviews of strategies to improve recruitment [4] and retention [13] into RCTs, personalisation of study invitations was not included. Further, to the best of our knowledge, no RCT has examined the personalisation of study invitations in the context of mental health research which is of particular importance given recruitment and retention to mental health trials has been identified as particularly challenging [18, 25].

### The ENGAGE host feasibility trial

Childhood cancer is a leading cause of death and disease burden among children, and their parents, often their primary source of support, are actively involved in the child’s care even years after treatment. Sub-groups of parents report mental health difficulties [26, 27] productivity losses [28], daily life restrictions, and an unmet need for psychological support [29] after end of treatment. However, there remains a lack of evidence-based interventions tailored to parents, with their needs commonly unmet. Additionally, parents report barriers to seeking support such as lack of time, guilt, and putting the child’s health first [30, 31]. In accordance with the Medical Research Council complex interventions framework [32, 33] we have conducted a series of studies informing the development of an internet-administered, guided, self-help programme, EJDeR (Swedish acronym). EJDeR was co-created with parent research partners and is based on low intensity cognitive behavioural therapy (LICBT) for parents of children treated for cancer [34]. Studies have included reviewing existing evidence [26], exploring negative and positive experiences [35], conceptualizing distress [36], participatory action research [37], a cross-sectional survey [38], and professional and public involvement [34]. Dependent on the parents’ main presenting difficulties, LICBT behavioral activation or worry management treatment protocols are used for the treatment of depression and generalised anxiety disorder. EJDeR is delivered via the U-CARE-portal (Portal), a web-based platform designed to deliver internet-administered LICBT interventions and support research. EJDeR is guided by e-therapists, with parents receiving an initial assessment via videoconferencing or telephone, weekly written messages via the Portal, and a mid-intervention booster session via videoconferencing or telephone. Participants are located across Sweden. EJDeR is designed to be accessed from computers and mobile devices, and participants can choose where to use it. All research activities in the ENGAGE host feasibility trial were carried out via the Portal, e-mail, or telephone by staff located at the Department of Women’s and Children’s Health, Uppsala University, Sweden. We have tested EJDeR and intended study procedures for a planned future RCT of the clinical efficacy and cost-effectiveness of EJDeR in the ENGAGE host feasibility trial [39] (ISRCTN 57233429).

### Aims and objectives

This study aimed to use a SWAT, embedded within the ENGAGE host feasibility trial, with the primary objective to investigate the effect of personalised versus non-personalised study invitation letters on recruitment rates. We further aimed to explore a number of secondary objectives, investigating the effect of personalised versus non-personalised study invitation letters on a number of secondary outcomes related to the recruitment and retention.

## Methods

This SWAT is reported in accordance with guidelines for reporting embedded recruitment trials [40]. The SWAT is registered in the ISRCTN registry (ISRCTN18404129) and the Northern Ireland Hub for Trials Methodology Research SWAT repository (SWAT 112). A full protocol for the SWAT has been published [24].

### Design

A parallel group embedded RCT with a 1:1 allocation ratio to investigate the effect of personalised compared with non-personalised study invitation letters on recruitment rates [24].

### Participants

Participants eligible for inclusion in the ENGAGE host feasibility trial were parents of children diagnosed with cancer during childhood (0–18 years) who completed cancer treatment 3 months to 5 years previously and had a self-reported need for psychological support. The full eligibility criteria are outlined in the ENGAGE host feasibility trial study protocol [39]. All potential participants who were invited via the Childhood Cancer Registry into the ENGAGE host feasibility trial were eligible for the SWAT.

### Recruitment

The ENGAGE host feasibility trial adopted two recruitment strategies: postal study invitation packs via the Swedish Childhood Cancer Registry (National Quality Registry) and advertisements on social media and patient organisation websites. Only participants recruited via the Childhood Cancer Registry were included in the SWAT. Children’s personal identification numbers were gathered from the Childhood Cancer Registry, and subsequently matched with parents’ names and addresses via the Swedish Tax Agency’s registry NAVET. Study invitation packs were sent in blocks of 100, every 30 days, until the target sample size of 50 participants was reached. Invitation packs included a study invitation letter, a study information sheet, a link to the study website (the Portal) and a reply slip with a stamped addressed envelope. Contact details to the research team were provided and parents were able to opt-out from further contact with the research team via post, telephone, e-mail, or the Portal. Opt-out forms included a ‘reasons for non-participation’ questionnaire. The use of an opt-out recruitment strategy was approved by the Regional Ethical Review Board in Uppsala, Sweden (Dnr: 2017/527). As parents were individually invited into the study, there was a possibility for two parents of the same child to be invited and enrolled into the trial.

### Interventions

Potential participants to the ENGAGE host feasibility trial were randomised to be invited via: a personalised study invitation letter, including name and address of the parent (intervention group) or a non-personalised study invitation letter not including name and address of the parent (control group). Invitations did not differ in any other aspect and translated versions can be found as a supplement to this paper, see Additional files 1 and 2.

### Study procedures

Potential participants could access study information, in text and video format, and provide consent via the Portal. Potential participants who wished to opt-out of the study could do so by completing an opt-out form and reasons for non-participation questionnaire via the Portal or by paper via post. Participants could also opt-out by telephoning or e-mailing the research team. Potential participants who wanted additional information from the research team before providing consent via the Portal could register interest via a postal reply slip included in the invitation pack, or by telephoning, or e-mailing the research team. Those registering interest were provided with study information by the research team and asked to provide consent via the Portal if interested in study participation. The eligibility interview (including the Mini-International Neuropsychiatric Interview (M.I.N.I. [41]), semi-structured interview at baseline, semi-structured interview at post-treatment (12 weeks), M.I.N.I. at post-treatment (12 weeks) and M.I.N.I. at 6-month follow-up was conducted over the telephone. Online Portal assessments at baseline, post-treatment, and 6-month follow-up were done over the Portal, or, if preferred by the participant, over telephone with a member of the research team. Reminders to complete online Portal assessments were provided if participants did not complete online Portal assessments within 2 weeks of gaining access. Participants who dropped out of the study were asked to provide a reason, however they were reminded that they did not need to report a reason if they preferred not to.

### Outcomes

The primary outcome was the proportion of participants in the intervention group and the control group enrolled into the ENGAGE host feasibility trial. Secondary outcomes were the proportion of potential participants invited into the study in each group that:

- Registered interest in participating in the ENGAGE host feasibility trial
- Opted out of the ENGAGE host feasibility trial
- Completed reasons for non-participation questionnaire in the ENGAGE host feasibility trial
- Consented to participate in the ENGAGE host feasibility trial
- Completed the eligibility interview for inclusion in the ENGAGE host feasibility trial
- Completed the semi-structured interview at baseline in the ENGAGE host feasibility trial
- Completed the online Portal assessment at baseline in the ENGAGE host feasibility trial
- Were retained at post-treatment (12 weeks) and 6-month follow-up in the ENGAGE host feasibility trial respectively i.e. a) completed the M.I.N.I. at post-treatment (12 weeks), b) completed the semi-structured interview at post-treatment (12 weeks), c) completed the online Portal assessment at post-treatment (12 weeks), d) completed the M.I.N.I. at 6-month follow-up, e) completed the online Portal assessment at 6-month follow-up
- Required a telephone reminder at baseline, post-treatment, and 6-month follow-up respectively in the ENGAGE host feasibility trial to complete the online Portal assessment

#### Protocol changes

The secondary outcome “consented to participate in the ENGAGE host feasibility trial” was added after the publication of the study protocol [24], but prior to statistical analysis of the data presented herein. The outcome “completed the semi-structured interview at baseline in the ENGAGE host feasibility trial” was added when the statistical analysis had commenced. In the SWAT protocol [24], retention outcomes were collapsed to include completion of all post-treatment (12 weeks) and 6-month follow-up assessments respectively. Due to different retention rates for different assessments, outcomes are reported separately. Online Portal assessments consist of clinical outcomes included in the ENGAGE host feasibility trial [39].

### Data collection

Study data collected outside of the Portal was entered onto paper-based case report forms and subsequently manually entered independently by two research assistants onto a Microsoft® Access database with data exported into Microsoft® Excel spreadsheets. Portal data was extracted by an in-house system developer and exported to Microsoft® Excel spreadsheets, with data prepared independently by two research assistants. Microsoft® Spreadsheet Compare was used to compare all data entries to identify discrepancies and missing values, with any discrepancies discussed and resolved in data management meetings.

### Sample size

The SWAT sample size was dependent on the ENGAGE host feasibility trial and therefore no sample size calculation was made. It was anticipated that 600 invitations would be needed to reach the target sample size of 50 in the ENGAGE host feasibility trial [39] which would have provided 90% power to identify a 7.5% difference between groups in recruitment rate at a two-sided alpha = 0.05 [24]. We randomized 509 potential participants into the SWAT, however, no post-hoc power analysis was conducted given the limitations of post hoc analysis, especially when reporting negative trial results [42].

### Randomisation

Eligible participants were randomised in a 1:1 ratio to the intervention group (personalised study invitation letter) or control group (non-personalised study invitation letter) using simple randomisation without stratification. To ensure allocation concealment, a member of the Portal development team, not involved in participant recruitment, produced a computer-generated randomised sequence outside of the Portal. The randomisation software was developed in C# and written specifically for randomisation into the SWAT and was designed to read a de-identified text file-list of potential participants and output the participants in two randomised groups into a CSV file. The participant allocation list was returned to the research team to implement. Participants were allocated a Recruitment ID number within the study invitation pack dependent on SWAT intervention allocation. Participants entered this Recruitment ID number when providing online consent, or opting out of the study, on the Portal. In addition, an allocation list with the participants’ personal identification number was stored on a secure USB in a locked filing cabinet. Only research staff members responsible for preparing and sending the invitation packs had access to the allocation list. To assure adherence to the randomisation sequence, a random sample of 10% of every 50 invitation letters to the ENGAGE host feasibility trial were checked for accuracy.

Eligible participants were not informed about the SWAT, and were therefore blind to the SWAT hypothesis and unaware they were participating in an embedded recruitment trial. It was not possible for research team members involved in sending study invitations, or working with recruitment to be blind to group allocation. However, the researcher conducting the statistical analysis (third author MÖ) was blind to group allocation. Additionally, each outcome variable name was allocated a letter (aaa-sss) and their order of presentation randomised in the dataset provided for statistical analysis, to further ensure blinding.

E-therapists who guided the EJDeR intervention were blind to group allocation in the SWAT, with the exception of one e-therapist (to five participants) who was also a member of the research team and thus had access to the information about SWAT group allocation.

### Statistical analysis

Statistical analyses were conducted on an intention-to-treat basis. A two-sided *p* value of < 0.05 was chosen to indicate statistical significance. A decision was made to use Stata (Stata/MP 16.1, StataCorp) instead of SPSS as stated in the study protocol, [24], as preferred by the researcher conducting all analyses (MÖ). Categorical outcomes were reported with numbers and percentages. Differences in proportions between groups for the primary and secondary outcomes were estimated using logistic regression, with the result reported as an odds ratio with 95% confidence interval and *p*-values. If two parents of the same child were enrolled in the ENGAGE host feasibility trial, this would cause some dependency in the data between the two parents. There were two cases whereby two parents of the same child were enrolled into the ENGAGE host feasibility trial who were invited via the Childhood Cancer Registry. In one case both parents were randomised into the intervention group (personalised study invitation letter). In the other case one parent was randomised to the intervention group and one to the control group (non-personalised study invitation letter).

In the original data analysis plan, Logistic regression models would include stratification by parent and child gender (male/female). However, due to ethical considerations, we were unable to use information on gender unless this data was reported to the research team (e.g., during eligibility interviews on or when opting out of the study). Subsequently, there was too little data on gender to stratify the analyses on gender.

### Public involvement

Procedures for the ENGAGE host feasibility trial were developed in collaboration with a parent research partner group consisting of two fathers and two mothers, aged 45–54, with lived experience of being a parent to a child treated for cancer. For the SWAT, parent research partners provided feedback on general wording of the invitation letters, and how to personalise the letter provided to the intervention group. Parent research partners were asked about preferences regarding personalising e.g., to include the child’s name along with the parent’s, or to only use the parent’s name. The group preferred to only include the parent’s name and advised that including the child’s name may potentially be considered an invasion of privacy [24].

## Results

### Recruitment

**Fig. 1:**
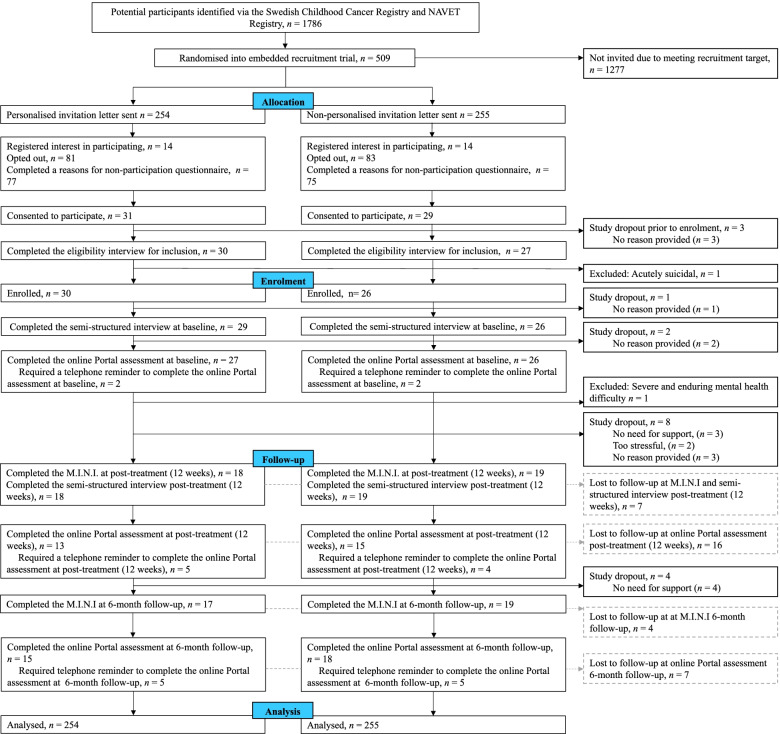
Study flow of study within a trial (SWAT) participants in the ENGAGE host feasibility trial. *Note*. Solid black lines denote participant flow through the study, including study drop outs i.e., those who discontinued the study. Dashed grey lines represent participants that were lost to follow-up during assessments at post-treatment (12 week) and 6-month follow-up respectively, but had not dropped out of the study

Recruitment via study invitation letters to the ENGAGE host feasibility trial took place between July 3rd and November 30th 2020. The recruitment target was met after 509 study invitations were sent. Post-treatment (12 weeks) data collection took place between September 22nd 2020 and April 8th 2021, and 6-month follow-up data collection between April 18th and October 4th 2021. See Fig. 1 for participant flow.

### Outcomes

Of the 509 potential participants invited, 56 (11.0%) were enrolled into the ENGAGE host feasibility trial: personalised: 30/254 (11.8%) and non-personalised: 26/255 (10.2%). No statistically significant effects on personalisation of enrolment were found (OR 1.18, 95% CI 0.68–2.06). No significant effects on personalisation were found for any of the secondary outcomes, see Table 1.

**Table 1:** Descriptive summaries and odds ratios for primary and secondary outcomes

Outcome	Total (*n* = 509)	Personalised (*n* = 254)	Non-personalised (*n* = 255)	Odds ratio	95% confidence interval		*p*
	*n* (%)	*n* (%)	*n* (%)		Lower threshold	Upper threshold	
Enrolled	56 (11.0)	30 (11.8)	26 (10.2)	1.18	0.68	2.06	0.56
Registered interest in participating	28 (5.5)	14 (5.5)	14 (5.5)	1.00	0.47	2.15	0.99
Opted out	164 (32.2)	81 (31.9)	83 (32.6)	0.97	0.67	1.41	0.87
Completed a reasons for non-participation questionnaire	152 (29.9)	77 (30.3)	75 (29.4)	1.04	0.71	1.53	0.82
Consented to participate	60 (11.8)	31 (12.2)	29 (11.4)	1.08	0.63	1.86	0.77
Completed the eligibility interview for inclusion	57 (11.2)	30 (11.8)	27 (10.6)	1.13	0.65	1.96	0.66
Completed the semi-structured interview at baseline	55 (10.8)	29 (11.4)	26 (10.2)	1.14	0.65	1.99	0.66
Completed the online Portal assessment at baseline	53 (10.4)	27 (10.6)	26 (10.2)	1.05	0.59	1.85	0.87
Completed the M.I.N.I. at post-treatment (12 weeks)	37 (7.3)	18 (7.1)	19 (7.5)	0.95	0.49	1.85	0.87
Completed the semi-structured interview at post-treatment (12 weeks)	37 (7.3)	18 (7.1)	19 (7.5)	0.95	0.49	1.85	0.87
Completed the online Portal assessment at post-treatment (12 weeks)	28 (5.5)	13 (5.1)	15 (5.9)	0.86	0.40	1.85	0.71
Completed the M.I.N.I at 6-month follow-up	36 (7.1)	17 (6.7)	19 (7.5)	0.89	0.45	1.76	0.74
Completed the online Portal assessment at 6-month follow-up	33 (6.5)	15 (5.9)	18 (7.1)	0.83	0.41	1.68	0.60
Required a telephone reminder to complete the online Portal assessment at baseline	4 (0.8)	2 (0.8)	2 (0.8)	1.00	0.14	7.18	1.00
Required a telephone reminder to complete the online Portal assessment at post-treatment (12 weeks)	9 (1.7)	5 (2.0)	4 (1.6)	1.26	0.33	4.75	0.73
Required telephone reminder to complete the online Portal assessment at 6-month follow-up	10 (2.0)	5 (2.0)	5 (2.0)	1.00	0.29	3.51	0.99

## Discussion

### Summary

The primary objective was to investigate the effect of personalised versus non-personalised study invitation letters on recruitment rates, i.e., rates of enrolment into a host trial examining the feasibility of the internet administered, guided, self-help programme, EJDeR, for parents of children treated for cancer. Personalisation of study invitations had no effect on enrolment in the host trial or any of the secondary outcomes. Numbers were larger in the intervention group (personalised study invitation letters: 30/254 [11.8%]) versus the control group (non-personalised study invitation letters: 26/255 [10.2%]) for rate of enrolment and the majority of secondary outcomes related to the recruitment and screening process i.e. consented to participate, and completed the eligibility interview, semi-structured interview, and online Portal assessments at baseline. The numbers for opting out of the study were smaller in the intervention group (81/254 [31.9%] than the control group (83/255 [32.6%]) and for registered interest in participating the numbers were the same in the intervention group (14/254 [5.5%] and the control group (14/255 [5.5%]. For outcomes related to retention the opposite trend is visible. At post-treatment (12 weeks) numbers completing were smaller in the intervention group (M.I.N.I. and semi-structured interview: 18/254 [7.1%]; online Portal assessment: 13/254 [5.1%]) than in the control group (M.I.N.I. and semi-structured interview: 19/255 [7.5%]; online Portal assessment: 15/255 [5.9%]). At 6 months follow-up numbers completing were also smaller in the intervention group (M.I.N.I.: 17/254 [6.7%]; online Portal assessment: 15/254 [5.9%]) than the control group (M.I.N.I.: 19/255 [7.5%]; online Portal assessment: 18/255 [7.1%]). However, given the wide confidence intervals for all primary and secondary outcomes relating to recruitment and retention, findings should be interpreted with caution.

### Limitations

First, although the ENGAGE host feasibility trial recruited to target, as a feasibility study, only a small number of potential participants were invited and subsequently recruited and retained. As such, the embedded recruitment trial may be underpowered to detect between group differences. Future embedded recruitment trials, within large-scale evaluation RCTs, are warranted to further investigate the effect of personalised versus non-personalised study invitation letters on recruitment and retention rates. Given the current lack of similar RCTs, a cumulative meta-analysis is not possible and further justifies the need to conduct further research [43]. Second, both the personalised and non-personalised study invitation letters contained elements that may be perceived as personalised. For example, names and addresses on envelopes for both groups were written by hand, and all invitation letters were signed by the principal investigator and a parent research partner. This could have lessened the effect of the intervention. Indeed, some evidence suggests handwriting the address on envelopes increases survey response rates [19]. In addition, it was not possible for the research team to know which given name potential participants used and subsequently middle names were included when personalising study invitation letter. Using both first and middle names could be perceived as less personal, further reducing the impact of the intervention. Third, stratification on gender in the logistic regression model was not possible as we could only include data on gender when reported to the research team (e.g., during eligibility interviews and when opting out of the study). However, our previous longitudinal research with the population has not found any significant differences between parents who participated in assessment completion at various time points, versus those who did not complete assessments, in relation to gender (parent and child) [44, 45]. Therefore, we consider not being able to stratify on gender in the logistic regression model unlikely to have impacted our results. Future studies may wish to seek ethical approval to report certain sociodemographic characteristics, where possible, for all participants approached and randomised into a SWAT in accordance with the guidelines for reporting embedded recruitment trials [40]. This would facilitate an examination of potential differences between participants and non-participants on certain demographic factors, such as gender, and enable more extensive analysis in the future, as well as providing important information concerning the generalisability of the SWAT results. In addition, we did not plan to report baseline characteristics, presented by SWAT group allocation, of those enrolled into the ENGAGE host feasibility trial, which would have provided further important contextual information. Finally, we did not apply for ethical approval to report how often two parents of the same child were randomised into the SWAT and on these occasions whether parents were allocated to different intervention groups. Of those parents enrolled into the ENGAGE host feasibility trial, in only one case were two parents of the same child allocated to different intervention groups. Therefore, the number of times this happened out of all parents randomised into the SWAT is considered likely to be small. It is also considered unlikely parents would be unblinded to the SWAT hypothesis, however future similar SWATs should look to implement processes to prevent two potential participants in the same household being allocated to different SWAT intervention groups.

### Strengths and interpretation of the findings in the context of the wider literature

Despite the aforementioned limitations, this study has important strengths. Research on recruitment strategies to clinical trials has been identified as much needed to increase the quality of clinical research and thus reduce research waste [7–12] and this study adds to the emerging body of evidence on the subject. We investigated the effect of personalised versus non-personalised study invitation letters on multiple outcomes related to both recruitment and retention, which, to the best of our knowledge, has not been done before. The methodology is straightforward and easy to undertake, and this study could be used as a template for future SWATs. In future studies, we recommend the use of electronic case report forms to facilitate data collection, since the use of paper-based case report forms was time consuming, and there is evidence to suggest paper-based case report forms are more prone to data entry errors, such as data omissions [46].

Current literature on effects of personalisation of study invitation letters on recruitment and retention is limited. The personalisation of study invitation letters has been found to have a positive effect on survey response rates [21–23]. However, our results are in line with a recent embedded recruitment trial that found a non-significant positive effect for the personalisation of study invitation letters on the recruitment of general practitioners [47]. Importantly, even small improvements in recruitment rates could be of benefit for clinical trial recruitment, especially considering the personalisation of study invitation letters is a pragmatic, feasible, and low-cost strategy. Another interesting finding in the current study was that, even if not statistically significant, data indicates that less participants in the personalised study invitation group were retained at follow-up e.g., completed assessments post-treatment (12 weeks) and at 6-months follow-up. Two recent studies have shown that personalisation of text message reminders is not associated with increased return rates of follow-up questionnaires within clinical trials [48, 49], whereas a further recent study found a favorable effect of personalised reminders via text messages [50]. However, to date very few studies have investigated the effect of personalised study invitations on secondary outcomes pertaining to retention.

## Conclusions

Personalisation of study invitations had little effect on recruitment, and a non-significant positive effect was found, with an enrolment rate of 11.8% (30/254) in the personalised group and 10.2% (26/255) in the non-personalised group. Given the small sample size, and lack of similar embedded recruitment RCTs, the effect of the personalisation of study invitations on recruitment and retention remains uncertain and there is a need to conduct similar SWATs within large-scale evaluation RCTs with different populations.

## Supplementary Information

**Additional file 1.**

**Additional file 2.**

## Data Availability

The research data supporting the findings of this study is stored in Zenodo repository with the identifier doi.org/10.5281/zenodo.5796065. Access to the data stored in Zenodo is available upon written request from the corresponding author.

## Abbreviations

LICBT: Low Intensity Cognitive-Behavioural Therapy
M.I.N.I.: Mini-International Neuropsychiatric Interview
RCT: Randomised Controlled Trial
SWAT: Study Within A Trial
