# Supplementary material for: The effect of personalised versus non-personalised study invitations on recruitment within the ENGAGE feasibility trial: an embedded randomised controlled recruitment trial
Source: BMC Med Res Methodol. 2022 Mar 6;22:65. doi: 10.1186/s12874-022-01553-5 (PMC8898447; doi:10.1186/s12874-022-01553-5)
Supplement: Supplementary file 1 — Additional file 1. [file 12874_2022_1553_MOESM1_ESM.pdf]

Note: This is an English translation of the original Swedish invitation letter

UPPSALA UNIVERSITET

ENGAGE

Invitation to the research project:

ENGAGE, testing of the self-help program EJDeR for parents of children previously treated for cancer

First Name Surname
Address
Postal code/City

Hej [insert parent's name]!

As a group of researchers at Uppsala University, we have developed an internet-based self-help program for parents of children previously treated for cancer. We have developed the program together with parents of children previously treated for cancer. We hope that the program can help parents who are experiencing emotional difficulties related to their child's illness, such as feeling low, down, sad, scared, worried, or angry.

You are invited to take part in a project in which we will test if parents wish to use the program and experience it as helpful. The sponsor is Uppsala University. We were provided with your information via the Swedish Child Cancer Registry and the Swedish Tax Agency's Registry NAVET.

In this invitation pack you will find:

- A full study information sheet
- A reply slip should you like to receive more information
- An opt-out form if you would rather not participate
- A stamped addressed envelope

You can also find more information about the project by:

- Visiting: www.u-care.se/Engage
- Emailing: foraldrar@kbh.uu.se
- Calling: 018-471 65 77

If you are interested in participating in the project, you can register via www.u-care.se/Engage using this the following recruitment code: [insert code]

Kind regards,

Louise von Essen
Professor, Principal Investigator
Department of Women's and Children's Health
Uppsala University
Tel: 070-425 07 14
Email: louise-von.essen@kbh.uu.se

Martin Hedqvist
Parent of a child previously treated for cancer

Our research is supported by:

CANCERFONDEN
BARNCANCER FONDEN
Vetenskapsrådet
